# Effect of Formation Route on the Mechanical Properties of the Polyethersulfone Composites Reinforced with Glass Fibers

**DOI:** 10.3390/polym11081364

**Published:** 2019-08-19

**Authors:** Galal Sherif, Dilyus Chukov, Victor Tcherdyntsev, Valerii Torokhov

**Affiliations:** Center of composite materials, National University of Science and Technology “MISIS”, Leninskii prosp, 119049 Moscow, Russia

**Keywords:** glass fibers, surface modification, polyethersulfone, impregnation

## Abstract

Interfacial interaction is one of the most important factors that affect the mechanical properties of the fiber reinforced composites. The effect of fabrics′ sizing removal from glass fibers’ surface by thermal treatment on the mechanical characteristics of polyethersulfone based composites at different fiber to polymer weight ratios was investigated. Three fiber to polymer weight ratios of 50/50, 60/40, and 70/30 were studied. Flexural and shear tests were carried out to illustrate the mechanical properties of the composites; the structure was studied using Fourier-transform infrared spectroscopy and scanning electron microscopy. It was shown that solution impregnation of glass fabrics with polyethersulfone before compression molding allows to achieve good mechanical properties of composites. The thermal treatment of glass fabrics before impregnation results in an increase in flexural and shear strength for all the composites due to the improvement of fiber–matrix interaction.

## 1. Introduction

Growing attention has been given to the improvement of polymeric composite properties, especially in regards to their high strength-to-weight ratio [1,2,3]. Polyethersulfone (PES) is a superior performance engineering plastic with a high glass transition temperature *T*_g_ of 225 °C and operating temperature up to 180 °C. Due to several advantages such as high toughness; ease to produce and form complex shapes; good tribological properties; high modulus and strength; perfect fatigue resistance and dimensional stability; as well as rich fire, chemical and radiation resistance; PES is exceedingly eligible as a high-temperature tribo-material to substitute metals or ceramics [4]. Nevertheless, the tribological and mechanical properties of PES have to be evolved to gain the requirements necessary for several sophisticated applications, such as aerospace, automotive, and microelectronics [5].

Since enhancing the mechanical properties of polymer composites is the major task, glass fibers (GF) are widely used as reinforcing material [4]. GF supply beneficial adhesion to the polymer matrix, perfect aesthetic quality, and revised strength of the resulting composites [6]. Because GF possess high mechanical properties, low weight ratios, suitable heat resistance, and have a very cheap cost, they are attractive as reinforcers for polymers [7]. Thermoplastic composites reinforced with glass fibers were paid great attention because of their wide applications in automotive, aerospace, and many engineering applications [8]. Several studies have been carried out to investigate the mechanical properties of thermoplastics reinforced with glass fibers [9,10,11,12,13,14,15,16,17,18,19,20]. It was found that the properties of the composites are affected by the fibers’ geometry, orientation, concentration, and by the nature of the fibers [13,15,16,17,21,22] in addition to the interfacial adhesion between the fiber and polymer [23,24,25], which plays a crucial role in the composites’ strength.

The interfacial adhesion is affected both by the raw fibers’ surface coating (sizing) and by any GF surface treatment or modification [26,27,28,29,30,31,32,33]. Sizing prevents the damage of fibers after producing, makes their use in manufacturing easier, protects fibers from the environmental impact [28], and enhances the composites’ properties in the case of using sizing compatible with the matrix material [28,34]. Sizing could be provided by various materials, such as silanes, epoxies, paraffin and other coupling agents; and it could be one or more from these materials, but the actual and accurate sizing formula remains the secret of manufacturers [28]. However, most of the composites are made from epoxy resin, and most of the commercial sizings are often of the same nature. These sizings have relatively low degradation temperatures (about 250 °C) which are much lower than the processing temperatures of the engineering plastics such as PES. This would inevitably cause the problem of the sizing being degraded under high processing temperature, leading to weakened interfacial adhesion. A lot of methods (electrochemical, chemical, thermal, grafting, coating, and discharge plasma treatments, etc.) have been elaborated to enhance the interfacial adhesion between GF and polymers [35,36,37]. In the present work, we investigate the effect of formation route in addition to fabrics’ sizing removal from GF surface on the structure and mechanical properties of PES based composites.

## 2. Materials and Methods 

### Preparation of PES Composites 

**Figure 1:**
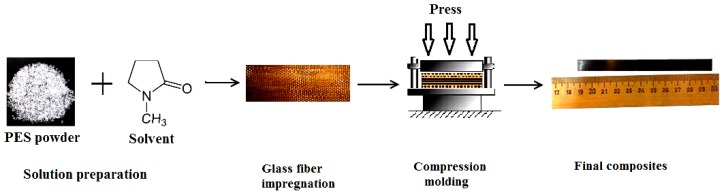
Preparation steps of polyethersulfone (PES) composites with compression molding.

Woven glass fabrics (NPO “Stekloplastic” Russia) (T-23 “260 ± 10 g/m^2^, 12 + 1 warp, 8 + 1 weft yarn/cm, 0.27 ± 0.03 thickness”) and PES Ultrason E2010 (Basf, Germany) powder were used as raw materials. A solvent of N-Methyl-2-pyrrolidone was used to produce PES solution to provide a good impregnation of fabrics with polymer. Solution formation was carried out with 20/80 polymer to solvent weight ratio for 24 h using a magnetic stirrer. Four routes were used for sample preparation: (1) compression molding of PES powder together with as-received glass fabrics; (2) compression molding of PES powder together with preheated glass fabrics; (3) compression molding of impregnated as-received glass fabrics with PES solution, and (4) compression molding of impregnated preheated glass fabrics with PES solution. The samples of PES solution impregnated glass fabrics were dried at 150 °C for 5 h to remove the solvent before compression molding. The bulk samples were prepared using the compression molding technique as shown in Figure 1. All samples were produced using the compression molding method at 350 °C and pressure of 10 MPa. The fiber to polymer weight ratio was varied as follows: 50/50, 60/40, and 70/30 wt %.

The glass fabrics were preheated using the furnace in air atmosphere at three temperatures (300, 350, and 400 °C) for 1 h and they were used to produce bulk samples which were tested to choose the best-preheated temperature.

Flexural and shear tests were performed to examine the PES-based composites using a Zwick/Roell Z020 universal test machine (Boston, MA, USA) equipped with 1 and 20 kN sensors and a contact strain measurement system MultiXtens. For flexural tests, samples of 110 mm × 10 mm × 2 mm and a span length of 80 mm (according to ISO 14125:1998) were prepared. Samples of 110 mm × 10 mm × 4 mm and a gauge length of 80 mm (according to ASTM D 3846) were prepared for shear tests. The flexural and shear tests were carried out at speeds of 10 and 1.3 mm/min, respectively, at room temperature. At least six samples were tested at each condition. 

FTIR spectroscopy of the samples after various treatments were obtained using a Nicolet 380 FT-IR spectrometer (spectral range of 4000–450 cm^−1^, resolution of 1 cm^−1^). A scanning electron microscope (VEGA 3 TESCAN) (Brno - Kohoutovice, Czech Republic) in a backscattered electron image mode was used to study the structure of the fracture surface of the composites. The samples were coated with a thin layer (10–15 nm) of carbon in a sputter coater. For the studies the composites′ fracture surface after flexural tests were used.

## 3. Results and Discussion

**Figure 2:**
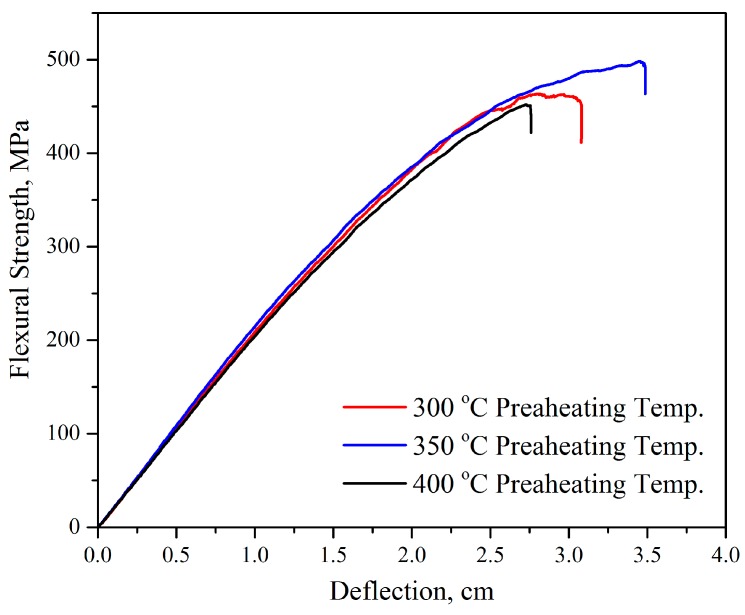
The strain–stress curves of 50/50 wt % composites prepared by route 4 using glass fibers (GF) preheated at various temperatures.

Elaboration of the advanced methods to improve the mechanical properties of the polymeric composites has become one of biggest challenges facing the industry recently. The effect of the preheating temperatures on the composites’ mechanical properties were studied. Figure 2 shows the effect of the fabrics which were preheated at different temperatures on the flexural strength of the composites. The composites were prepared by the compression molding of impregnated fabrics with 50/50 wt %. It can be noticed that the deflections were affected by the temperature, and were higher (3.5 cm) in case of 350 °C compared with 3 and 2.7 cm for 300 and 400 °C preheating temperature, respectively. In the case of 300 °C it seems that it was not enough to remove all the sizing coating which affected the adhesion between fibers and polymer, so the failure strain was less than in the 350 °C samples, while in the 400 °C samples the fiber strength decreased because of the heating and made the samples weaker [28]. It is seen that flexural strength was higher in case of GF preheating temperature of 350 °C (501 MPa) compared with 300 °C (450 MPa), and 400 °C (440 MPa). This trend was similar to that observed by many researchers [38,39,40,41] who studied the influence of heating the glass fiber. The results showed that if the fibers are heated above 400 °C, their strength drops rapidly. Taking into account these results, we used GF heated at 350 °C in further study.

**Figure 3:**
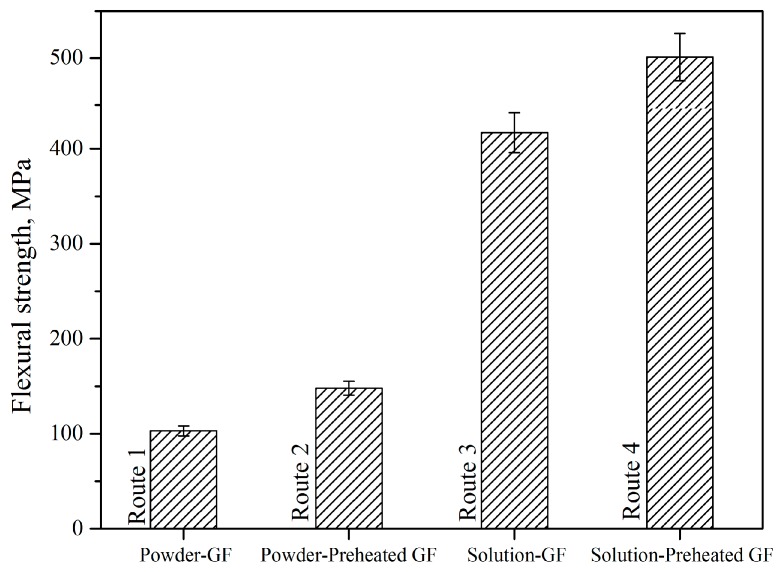
Flexural strength of different types of PES composites (50/50%, 350 °C preheating temperature).

Four types of composites (PES powder with untreated glass fibers (as-received) (route 1), PES powder with preheated glass fibers (route 2), solution impregnated PES with untreated glass fibers (route 3), and PES solution with preheated glass fibers (route 4) were examined to choose the optimum conditions for producing the samples. The composites prepared with 50/50 fiber to polymer weight ratio and the GF preheating temperature of 350 °C were studied. Figure 3 shows flexural strength magnitude depending on the composite’s formation route. As it is seen, the highest value of flexural strength was observed for sample obtained by PES solution impregnated preheated glass fabrics. The flexural strength of this composite was 501 MPa, which was significantly higher than values of 103, 148, 417 MPa for PES powder with as-received glass fabrics, PES powder with preheated glass fabrics, and PES solution with as-received glass fabrics samples, respectively. It can be concluded that PES in the solution form provides a good impregnation with the fabrics, and it was better with preheated fabrics. The presence of the solution enhanced the wettability and adsorption, which led to a good interfacial force that results in increased strength [42]. 

**Figure 4:**
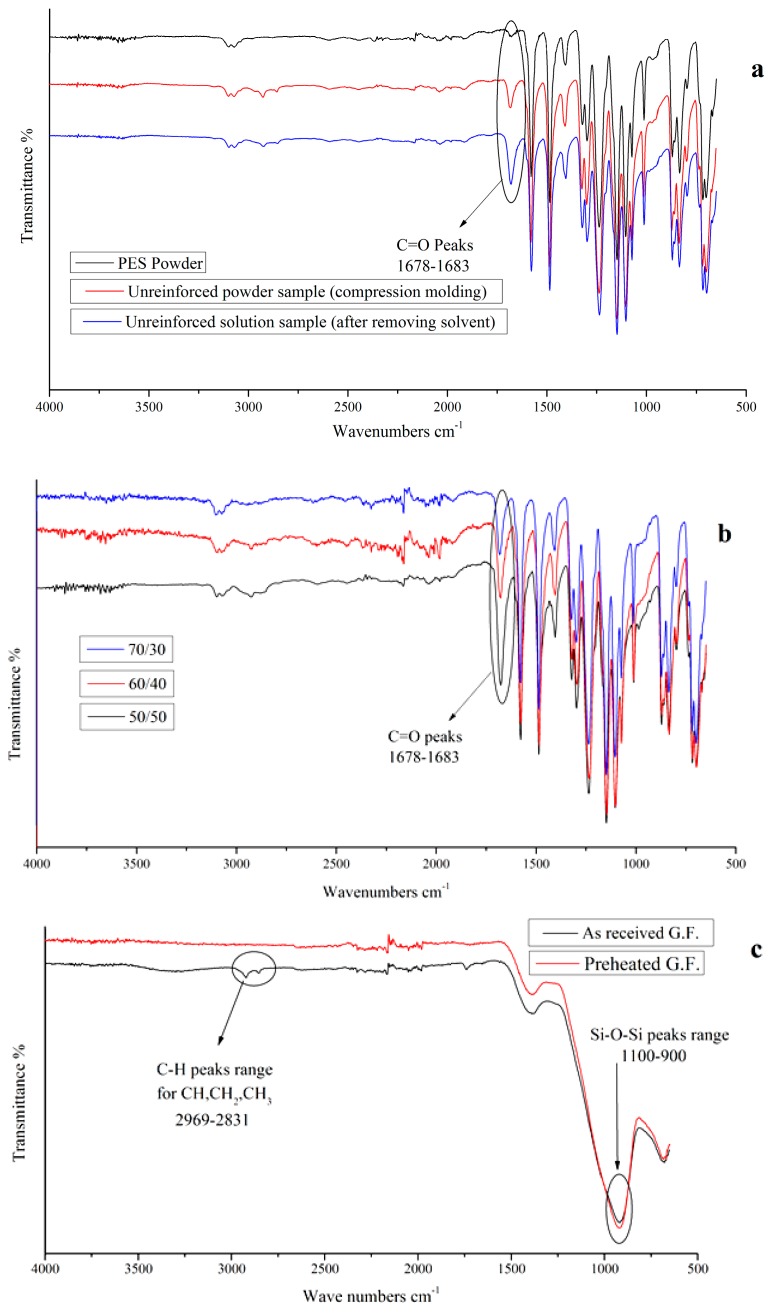
FTIR spectra for (**a**) initial PES powder, unreinforced PES powder sample after compression molding, and unreinforced solution sample after removing solvent. (**b**) PES impregnated composites (route 4) with different fiber to polymer weight ratios. (**c**) As-received GF and GF preheated at 350 °C.

FTIR spectra were carried out for characterization of the composites during the different stages of production. Figure 4a illustrates the characterization of the PES powder before any processing, PES samples prepared from PES powder by compression molding, and PES sample prepared from the PES solution after removing the solvent by heating at 150 °C for 5 h. From the results, it can be noticed that the major change is the amplitude of the C=O peaks (1678–1683) cm^−1^. It was found that the amplitude increased after compression molding due to the oxidation occurred during the heating, and in the case of the sample of PES in solution form, the presence of the small amount of solvent which contains C=O band makes the amplitude increases in the sample. Figure 4b shows the FTIR spectra of the PES composites with different fiber to polymer weight ratios (50/50, 60/40, and 70/30%). The 50/50 sample shows a higher C=O peak amplitude because the amount of the solvent is more than those in the other samples as a result of higher amount of polymer in 50/50 samples compared with other samples. The characterization of the as-received and preheated glass fabrics using FTIR is shown in Figure 4c. Since the silicone oxide band (1100–900 cm^−1^) in the glass fiber is a strong band, any band below 1200 cm^−1^ will not appear because of the strong absorption of the silicone oxide band. In addition, the as-received glass fabrics spectra shows noticeable peaks around 2969–2831 cm^−1^ which referred to stretching in C–H of CH, CH_2_, and CH_3_; while the spectra of preheated glass fabrics did not show any noticeable peaks before 2200 cm^−1^ which means that the sizing coating after heating was below the detection limit [29]. So, we can expect that almost of the sizing coating was removed.

**Figure 5:**
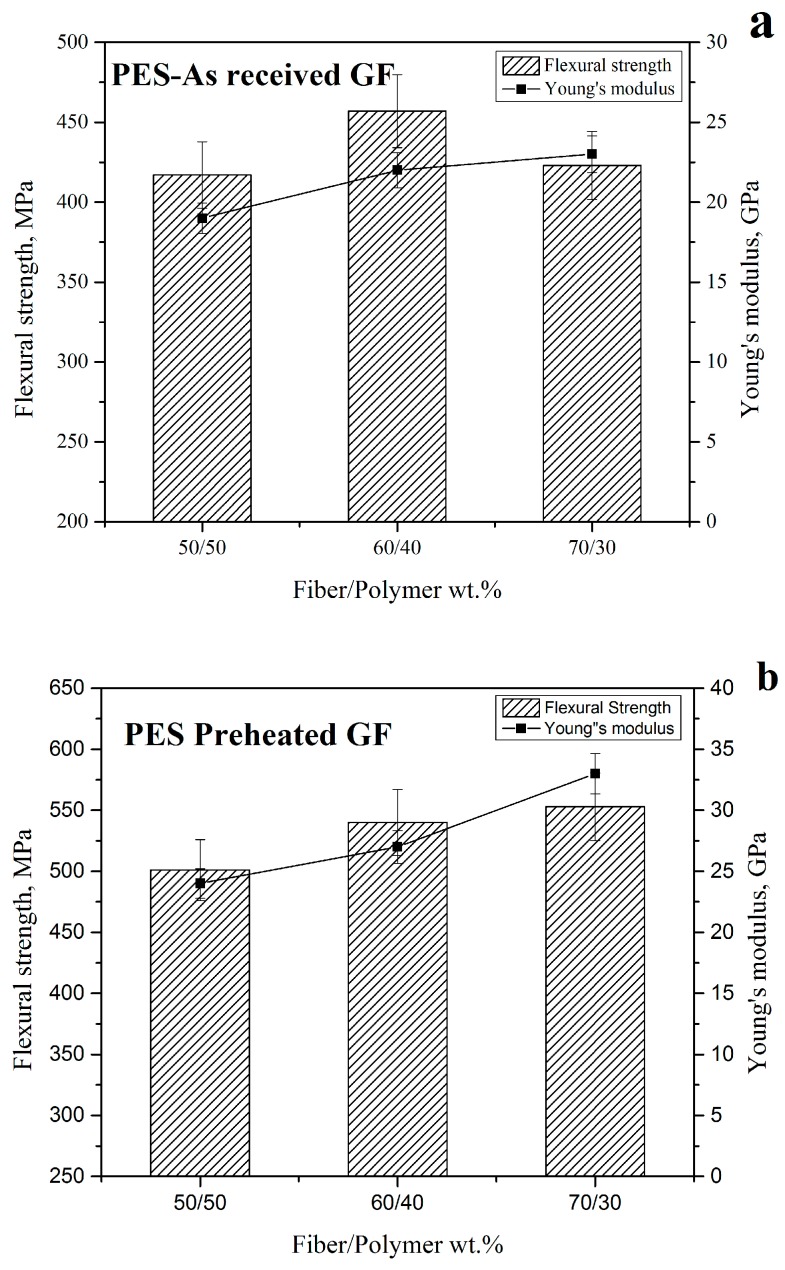
Flexural strength and Young’s modulus for composites reinforced with (**a**) as-received GF (route 3) and (**b**) preheated at 350 °C GF (route 4).

The comparison of the flexural strength and Young′s modulus of the PES composites reinforced with as-received and preheated GF is shown in Figure 5. For samples reinforced with as-received GF (Figure 5a) the highest value of flexural strength was observed for samples with the fiber to polymer ratio of 60/40, whereas for samples filled with preheated GF (Figure 5b) flexural strength nearly not depend on sample composition. Young’s modulus value gradually increases with an increase in the GF content for PES filled both with as-received and preheated GF. It can be proposed that in case of PES filled with as-received GF, an increase in the fiber amount affected the interface between the fiber and the polymer in the case of 70/30 samples which leads to flexural strength decrease and the fiber being pulled out from the matrix, as it will be shown in SEM images (Figure 7e). The problem of the poor interface was solved by preheating the fiber before using, which leads to removal of the sizing coating and enhanced adhesion between the fibers and polymer. It can be concluded that in addition to the increase in the properties of the composites due to the increase of the fiber content, the interfacial interaction between the fiber and the polymer was improved. This improvement of the adhesion between fiber and matrix provides a good stress transfer, which controls the strength of the composites [42,43].

**Figure 6:**
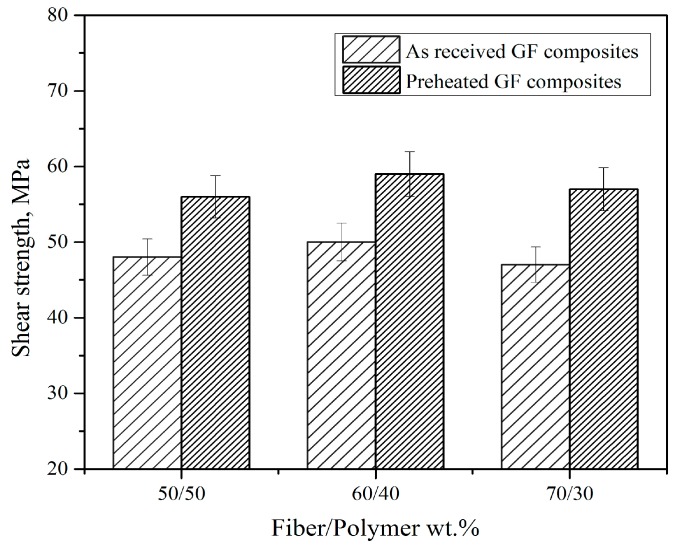
Shear strength of composites reinforced with as-received GF (route 3) and preheated at 350 °C GF (route 4).

To clarify this interfacing behavior, a shear test was carried out for the as-received and preheated GF reinforced composites to study the effect of preheating on the interfacial interaction. Figure 6 shows the shear strength for as-received and preheated GF reinforced composites. As shown in the figure the shear stress for the as-received glass fabrics composites increases from 48 MPa (for 50/50 samples) to 60 MPa (for 60/40 samples), while the shear strength in 70/30 samples (47 MPa) was affected by the decrease in polymer’s content which bond the fabrics layers together and directly affect the shear stress. On the other hand, a clear enhancement was noted in the preheated samples (56, 59, and 57 MPa for 50/50, 60/40, and 70/30 samples, respectively) compared with the as-received samples. It can be concluded that the shear strength increases in the case of preheated GF reinforced composites, which was an indication of the improvement of the interfacial interaction which leads to the increase of the flexural strength of the preheated composites compared with the as-received composites.

The improvement of the flexural strength and Young’s modulus for as-received and preheated glass fibers can be concluded from Table 1. It can be noticed that the flexural strength and Young′s modulus increased when the fiber percentage increased, which behaved like most of the thermoplastic composites [2,4,44]. The thermal treatment showed an additional improvement of all the samples [36], 20.1, 18.2, and 30.7% were the improvement percentages in flexural strength for the preheated composites for 50/50, 60/40, and 70/30, respectively, compared with the as-received composites and the improvement percentages in Young′s modulus were 26.3, 22.7, and 43.5%, respectively.

**Figure 7:**
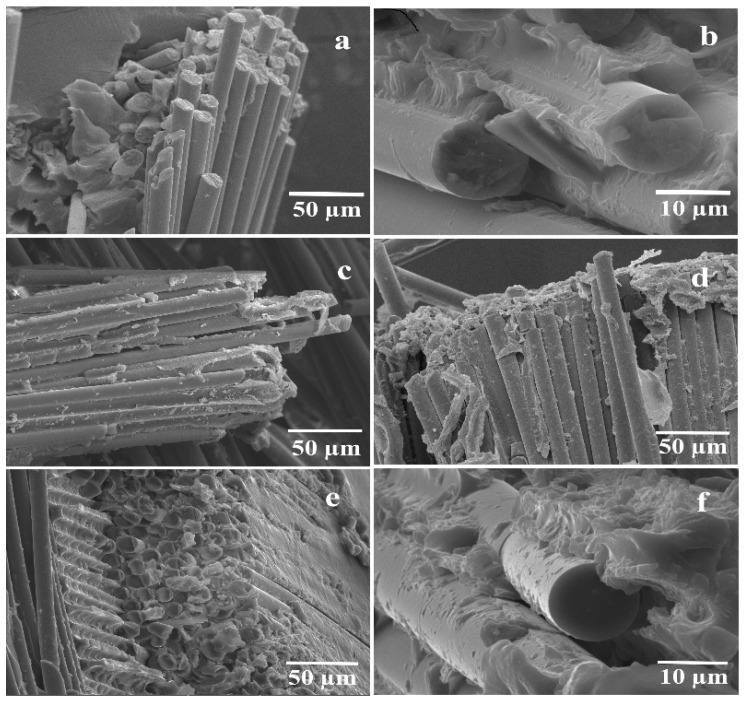
The structure of the fracture surface of the composites: (**a**) 50/50 as-received GF; (**b**) 50/50 preheated GF; (**c**) 60/40 as-received GF; (**d**) 60/40 preheated GF; (**e**) 70/30 as-received GF, and (**f**) 70/30 preheated GF composites.

SEM images of the fracture surfaces of the PES filled with 50/50 as-received GF, 50/50 preheated GF, 70/30 preheated GF, and 70/30 as-received GF are shown in Figure 7. The preheating of fibers allowed a good impregnation in the 50/50 sample as shown in Figure 7b compared with the sample 50/50 as-received GF in Figure 7a. The large amount of PES particles in preheated samples referred to the good interfacing between the fiber and polymer while a small amount of PES particles on the surface of the filaments of the as-received sample indicates bad adhesion. This leakage of adhesion led to fiber pull-out that appears in as-received samples. The 60/40 as-received composites shown in Figure 7c show a good distribution of PES particles on the glass fibers’ surface, these particles increased in amount and size in the case of 60/40 preheated composites shown in Figure 7d. This improvement was because of the good interface due to removing the sizing coating (in case of using preheated GF). However, the amount of PES particles decreased in the case of 70/30 preheated samples shown in Figure 7f but still provided a good impregnation which appears on the fibers’ surface. On the contrary, a poor adhesion occurred in the 70/30 as-received glass fibers reinforced composites (Figure 7e), which was evident in the form of fiber pull-out phenomenon.

## 4. Conclusions

A new method to improve the mechanical properties of PES composites by preheating the glass fibers to remove the sizing coating was suggested. Firstly, it was found that the method used to produce the samples has a great effect on the properties of the prepared composites. Using the PES in the solution form provides a good impregnation between the fibers and the polymer which improves the interaction between matrix and reinforcement resulting composite with better properties. The influence of the fiber to polymer ratio in addition to the effect of the thermal treatment of the fiber on the mechanical behavior of the composites was investigated. The results show that the flexural strength of the composites significantly increased when using the PES solution to impregnate the GF and preheated glass fabrics as reinforcements. The composites reinforced with as-received glass fabrics showed insufficient interfacial interaction between fibers and polymer, which resulted in lower mechanical properties compared with preheated composites. In the as-received composites 60/60 fiber to polymer ratio showed good results. The results reveal that the mechanical properties increased with increasing the fiber to polymer ratio, and the 70/30 samples were the best composition in case of using the preheated GF. Improvement was noticed in the mechanical properties of the PES-based composites due to the heat treatment of the glass fibers before using, which led to remove most of the sizing coating of fabrics (according to the FTIR of the fibers after preheating) and enhance the adhesion between the fabrics and polymer. According to SEM images, a good interface occurred in the case of using the preheated glass fabrics compared with those in as-received composites.

## Figures and Tables

**Table 1:** The flexural strength and Young’s modulus for composites reinforced with as-received GF (route 3) and preheated at 350 °C GF (route 4).

Fiber/Polymer	50/50		60/40		70/30	
Property	Flexural Strength, MPa	Young′s Modulus, GPa	Flexural Strength, MPa	Young′s Modulus, GPa	Flexural Strength, MPa	Young′s Modulus, GPa
As-received GF composites	417	19	457	22	423	23
Preheated GF composites	501	24	540	27	553	33
Increase, %	20.1	26.3	18.2	22.7	30.7	43.5
